# Recent Advances in and Challenges with Fe-Based Metallic Glasses for Catalytic Efficiency: Environment and Energy Fields

**DOI:** 10.3390/ma17122922

**Published:** 2024-06-14

**Authors:** Qi Chen, Zhigang Qi, Zhaoxuan Wang, Ziqi Song, Weimin Wang

**Affiliations:** Key Laboratory for Liquid-Solid Structural Evolution and Processing of Materials (Ministry of Education), School of Materials Science and Engineering, Shandong University, Jinan 250061, China; drqichen@163.com (Q.C.); qzg13019700233@163.com (Z.Q.); 202114121@mail.sdu.edu.cn (Z.W.); 202234218@mail.sdu.edu.cn (Z.S.)

**Keywords:** metallic glass, wastewater treatment, dye degradation, water splitting, catalytic activity

## Abstract

Metallic glass is being gradually recognized for its unique disordered atomic configuration and excellent catalytic activity, so is of great significance in the field of catalysis. Recent reports have demonstrated that Fe-based metallic glass, as a competitive new catalyst, has good catalytic activity for the fields of environment and energy, including high catalytic efficiency and stability. This review introduces the latest developments in metallic glasses with various atomic components and their excellent catalytic properties as catalysts. In this article, the influence of Fe-based metallic glass catalysts on the catalytic activity of dye wastewater treatment and water-splitting is discussed. The catalytic performance in different atomic composition systems and different water environment systems, and the preparation parameters to improve the surface activity of catalysts, are reviewed. This review also describes several prospects in the future development and practical application of Fe-based metallic glass catalysts and provides a new reference for the synthesis of novel catalysts.

## 1. Introduction

Dye wastewater is an important component of industrial wastewater. With the gradual development of science and technology, while using intelligent technology to enhance printing and dyeing efficiency, the increase in printing and dyeing wastewater discharge has attracted widespread attention [1]. Dyes are widely used in the dyeing of silk, wool, leather, paper, and textiles and can be used for biological dyeing. However, the direct discharge of dye wastewater into the environment causes serious water pollution [2], negatively impacting the living environments of human beings and making the problem of fresh water resources more serious. Dye wastewater has become a pollutant that must be degraded as quickly as possible in industrial wastewater because of its many forms, high toxicity, and being difficult to degrade [3]. In addition to the pollution of water environments, the energy problem is also a serious challenge that humankind has been facing. In recent years, many scholars have found that water splitting is a production technology that can be sustainably used to produce hydrogen and oxygen, and hydrogen is considered to be an effective alternative to traditional fossil fuels [4,5,6]. The whole water-splitting process consists of two half reactions, namely, the hydrogen evolution reaction (HER) on the cathode and the oxygen evolution reaction (OER) on the anode [7].

Metallic glass is another substance that is categorized as a metallic crystal. Metallic glass rapidly solidifies: atoms do not have the time to form an orderly crystal arrangement, instead forming a metastable structure comprising long-distance disordered and short-range ordered solid metal atoms [8]. In recent years, many scholars have found that metallic glass has a high density of low coordination, high surface residual stress, and a large number of unsaturated sites, so is considered to be the most competitive new catalyst in the field of dye wastewater treatment and water splitting [9]. The metallic glass systems currently reported for the catalytic degradation of dye wastewater mainly include Mg- [10,11], Al- [12,13], Co- [14], and Fe-based [15,16,17,18] systems. Fe-based metallic glass is considered by many scholars to be the most valuable catalyst for water environments [19]. Meanwhile, Wang et al. found that Fe powder can catalyze the degradation of dye wastewater but at a lower rate than that of metallic glass [20]. Moreover, in the field of water splitting, researchers have mostly used precious metal components as catalysts [21,22]. For example, Pd_40_Ni_10_Cu_30_P_20_ metallic glass has excellent self-stabilization properties and can also maintain 100% efficiency after 40,000 s of electrocatalytic testing [23]. Ir_25_Ni_33_Ta_42_ metallic glass nanofilm provides a large number of active sites, which greatly improves its electrochemical activity [24]. Ni_40_Zr_40_Ti_17_Pt_3_ metallic glass ribbon exhibits a small overpotential and low Tafel slope in the HER [25]. Meanwhile, TiO_2_ and ZnO commercial catalysts have also shown high catalytic activity in water-splitting studies [26,27]. However, the high cost of precious metals has been an important reason for the inability to industrialize these materials. Therefore, some scholars have used of nonnoble metals to prepare Fe-based metallic glass, Fe_50_Ni_30_P_13_C_7_, which can be directly used as a dual-function water-splitting electrode that shows high catalytic activity [28].

In order to inspire more scholars to develop and apply metallic glass catalysts, in this article, metallic glass preparation methods and applications of metallic glass as a catalyst in the environmental and energy fields in recent years are reviewed. In this review, the common methods for preparing amorphous alloys are introduced, which generally include melt spinning, ball milling and gas atomization. The mechanisms of the Fenton-like reaction system and persulfate system are described as catalysts for the degradation of dye wastewater. In the energy field, the efficiencies and mechanisms of the HER and OER with different catalysts during water splitting are introduced in detail. In addition, the possible problems facing the research on metallic glass catalysts in the future are pointed out, and some development directions are proposed, hoping to provide insights into the design of a new generation of industrial catalysts.

## 2. Metallic Glass Preparation Methods

Amorphous alloy is a substance known as a crystalline alloy. Due to ultra-quenching solidification, the atoms of the alloy do not arrange or crystallize in an orderly manner during solidification, and the obtained solid alloy atoms present a metastable structure with long-range disordered and short-range order called amorphous alloy [29,30]. Amorphous alloys have been developed for decades: In 1934, the German scientist Kramer was the first to use the evaporation deposition method and found that a metal film on a cold substrate of glass had different properties from crystal alloys [31]. In 1947, the American scientist Brenner prepared Ni-P amorphous alloy film using electrodeposition technology [32], and, in 1960, Duwez, a scientist at the California Institute of Technology, prepared thin ribbons of Au-Si and Au-Ge amorphous alloys via the rapid cooling of liquid metal [33]. In the following decades, many researchers began to prepare amorphous alloys via rapid solidification. Other methods for preparing amorphous alloys were also proposed [34]. In the past few decades, from the basic theory of their formation [35,36] to the their structure and properties [37,38], amorphous alloys have attracted wide attention in various research fields. In recent years, as a catalyst material, amorphous alloys have been a research hotspot in the fields of environment and energy [39,40]. Thus, in this section, we review the melt-spinning [41], ball-milling, and gas-atomization [20,42] methods used for preparing metallic glass catalysts. Table 1 summarizes some advantages and disadvantages of the three preparation methods.

### 2.1. Melt-Spinning Method

**Figure 1:**
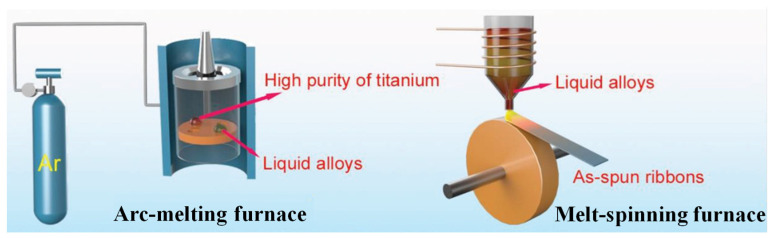
Master alloy-melting and melt-spinning process for ribbon amorphous alloys. Reproduced with permission [47]. Copyright 2019 WILEY-VCH Verlag GmbH & Co. KGaA, Weinheim, Germany.

Generally, only amorphous alloy ribbons can be obtained via melt spinning. This method requires a large cooling rate to promote the rapid solidification of the liquid alloy. The single roll strip method is generally used to prepare amorphous alloy ribbons [45]; the specific operation steps are as follows: First, the melted alloy ingots are placed in a quartz tube, and the bottom end of the quartz tube is kept at a suitable distance from the surface of the copper roll to ensure that there is no collision during the ribbon rejection process. Secondly, depending on the composition of the amorphous alloy prepared, the ribbon can be produced either in air or in a vacuum and an environment full of inert gases. And, the different components of the preparation of amorphous ribbon required cooling different rates; generally, the cooling speed should be between 10^5^ and 10^9^ K s^−1^. Finally, the alloy ingot in the quartz tube is heated and melted using an induction coil, and then the molten alloy is sprayed onto the surface of a high-speed rotating copper roller with argon gas, thus producing continuous amorphous alloy ribbons, as shown in Figure 1. Preparing uniform and continuous amorphous alloy with this technical method is relatively easy: the prepared amorphous alloy toughness is good and it can be used in a wide range of applications, but this method can only be used to prepare ribbon amorphous alloy. In catalytic research, the ribbons have exhibited high efficiency, sustainability, and reusability [46].

### 2.2. Ball Milling and Gas Atomization

**Figure 2:**
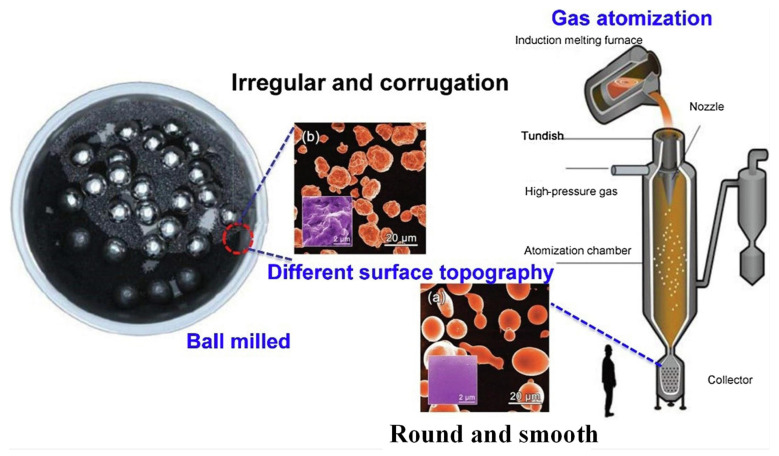
Ball-milling and gas-atomization methods to produce amorphous alloy powder (insets are the SEM images of both (**b**) ball-milled and (**a**) gas-atomized powder. Reproduced with permission [20]. Copyright 2012 WILEY-VCH Verlag GmbH & Co. KGaA, Weinheim, Germany). Reproduced with permission [19]. Copyright 2019 Elsevier Ltd.

Figure 2 shows the ball-milling (BM) and gas-atomization (GA) processes for manufacturing metallic glass powder. The SEM images show that the metallic glass powders produced via these two methods have different surface morphologies. The surface of ball-milled powder is rough, while the surface of GA-produced powder is smooth [20]. Ball milling is a kind of mechanical alloying method, relying on the vibration and rotation of the ball, so that the powder and the steel ball effectively collide, the powder is repeatedly broken and cold welded, and then the ideal amorphous powder is produced [48,49]. The size of the amorphous powder prepared via this method is generally between 100 nm and 50 μm, and it has good fluidity and usability. The equipment used for mechanical alloying is simple, the requirements for alloy composition are not strict, and amorphous powders of various shapes and sizes can be prepared according to the actual requirements. 

Gas atomization is another method used to prepare high-quality glass powder. The prepared master alloy is heated to form a molten metal liquid, which is then dropped into an atomization chamber and protected by argon or nitrogen and quickly cooled to a glassy state. The particle sizes of both manufactured powders are evenly distributed and exhibit enhanced catalytic properties in the treatment of water compared to those of commercial crystalline iron powders [20]. More importantly, in practical applications, the surface of ball-milled powder is rough, its specific surface area is larger, and it has higher catalytic efficiency than GA powder.

## 3. Catalytic Performance Evaluation

**Figure 3:**
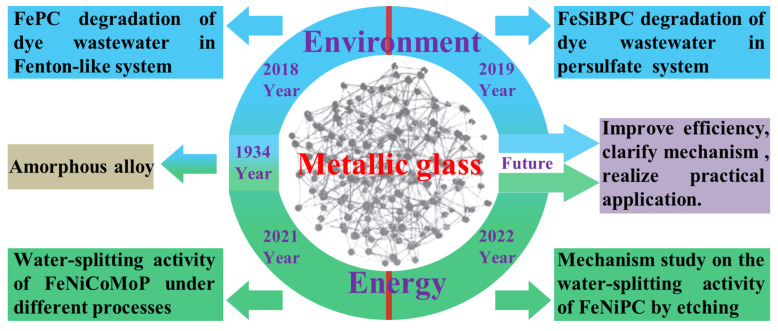
A representative diagram of the development history of metallic glass catalysts in the environment and energy fields.

In this article, the representative studies on dye wastewater treatment using Fe-based metallic glass as the catalyst in the environmental field and water splitting in the energy field are reviewed (Figure 3). In the treatment of dye wastewater, the research on catalysts in Fenton-like systems and persulfate systems is reviewed. In the energy field, the effects of different preparation processes on the catalytic activity of the catalysts are discussed. At the same time, the surface structure of the catalyst is calculated with a theoretical model, and the catalytic activity is further explained rationally.

### 3.1. Environmental Catalysts

**Figure 4:**
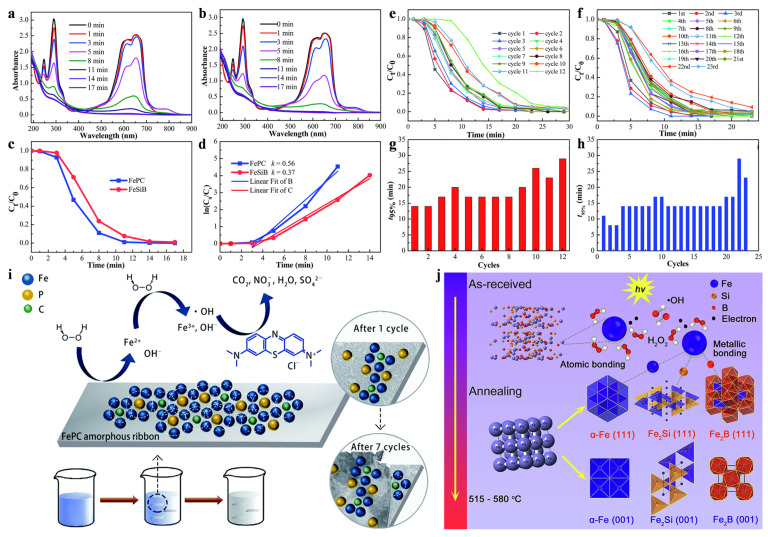
UV–Vis spectra of MB solutions using (**a**) FeSiB and (**b**) FePC. (**c**) Normalized concentration of MB solutions. (**d**) ln(*C*_t_/*C*_0_) vs. time curves. (**e**) Normalized concentration of MB solutions using FeSiB ribbons from the 1st to the 12th cycle. (**f**) Normalized concentration of MB solutions using FePC ribbons from the 1st to the 23rd cycle. The time required for 95% completion of the degradation process vs. reaction cycles for (**g**) FeSiB and (**h**) FePC ribbons. (**i**) Schematic diagrams of the pathway of MB degradation using FePC. Reproduced with permission [51]. Copyright 2019. The Royal Society of Chemistry. (**j**) Proposed catalytic mechanism of metallic glass on H_2_O_2_ activation. Reproduced with permission [35]. Copyright 2017. WILEY-VCH Verlag GmbH & Co. KGaA, Weinheim, Germany.

The dye color removal rate is one of the important indices used to characterize catalytic efficiency. Generally, in organic dyes, chromophores and auxochromes are two important components of the color rendering index. After adding Fe_78_Si_9_B_13_ (FeSiB) and Fe_80_P_13_C_7_ (FePC) ribbons to a reaction batch, the resulting UV–Vis absorption spectra of methylene blue (MB) solution over a series of time intervals are as shown in Figure 4a,b. The peaks at 618 and 653 nm represent chromophores and chromophores [50]. Note that as the reaction time increases, the two peaks gradually become invisible. This result suggests that chromophores are rapidly degraded, which may lead to eventual dye decolorization. The peak at 653 nm represents the chromogenic substance and therefore the normalized concentration peak as a MB solution, as shown in Figure 4c.

The degradation of MO solution by the two amorphous ribbons remained basically unchanged in the first 3 min and then decreased rapidly. The equation of the kinetic model is [50]

ln(*C*_0_/*C*_t_) = *kt* (1)

where *k* is the reaction rate constant. ln(*C*_0_/*C*_t_) vs. *t* curves are shown in the upper inset in Figure 4d. The *k* value of the FePC ribbon is greater than that of the FeSiB ribbon. Obviously, FePC ribbon has a better decolorization effect on the MB solution than FeSiB ribbon.

Reusability is very important in evaluating the wastewater remediation potential of amorphous ribbons. The FeSiB amorphous ribbon synthesized via the Fenton-like reaction can decompose 95% of the MB within 17 min in eight cycles, as shown in Figure 4e,g. There is a small drift in the fourth cycle, but in the ninth cycle, the solution needs more than 20 min to complete the degradation. However, the synthetic FePC ribbon is able to degrade 95% of the MB within 14 min of 19 cycles in a Fenton-like reaction, as shown in Figure 4f,h. Obviously, FePC amorphous ribbon has better reusability than FeSiB amorphous ribbon. By analyzing the microstructure of FePC during MB degradation and cyclic tests, the Fenton-like reaction pathway can be drawn, which is shown in Figure 4i. FePC ribbons were added to an MB solution containing H_2_O_2_ and hydrochloric acid, and metal iron atoms reacted with the H_2_O_2_ to form strongly oxidizing ^•^OH groups on the surface of the ribbons. The MB molecules then thoroughly oxidized and broke down into small molecules. During the reuse process, the continuous oxidation of iron in the FePC causes the surface to form a three-dimensional nanopore structure, which then transforms into a cotton-like structure. The cotton-like structure mainly contains the elements P, C, and O, and a small amount of iron. Mechanical stirring gradually removes the loose cotton layer from the ribbon, exposing the “fresh” FePC surface below to the MB solution, which prevents the degradation of the FePC ribbon. Finally, the effects of the metallic glass and annealed relaxation structure in Fenton-like reaction is summarized in Figure 4j. Compared with those of partially crystalline amorphous alloys, one of the most critical properties of metallic glass is the increased chemical activity, as it is more likely to activate the electrons around randomly disordered atoms with weak atomic bonding structures [41]. The high mobility of the electrons greatly improves the efficiency of dye degradation [51]. The process of Fe-based metallic glass catalytic degradation of dye wastewater through Fenton-like reaction can be generally divided into three steps [52]:

Fe^0^ + H_2_O_2_ → Fe^2+^ + 2OH^−^ (2)

Fe^2+^ + H_2_O_2_ → Fe^3+^ + ^•^OH + OH^−^ (3)

^•^OH + Organics → Products (4)

**Figure 5:**
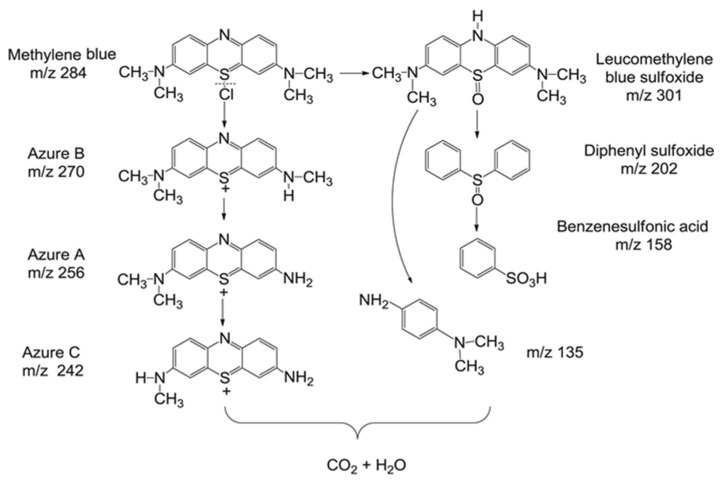
Proposed degradation pathways of MB by ^•^OH radicals. Reproduced with permission [53]. Copyright 2018 Elsevier Ltd.

Figure 5 shows the degradation pathways of MB dye under the action of ^•^OH groups: it is first decomposed into small molecular organic matter and then completely mineralized into carbon dioxide and water.

**Figure 6:**
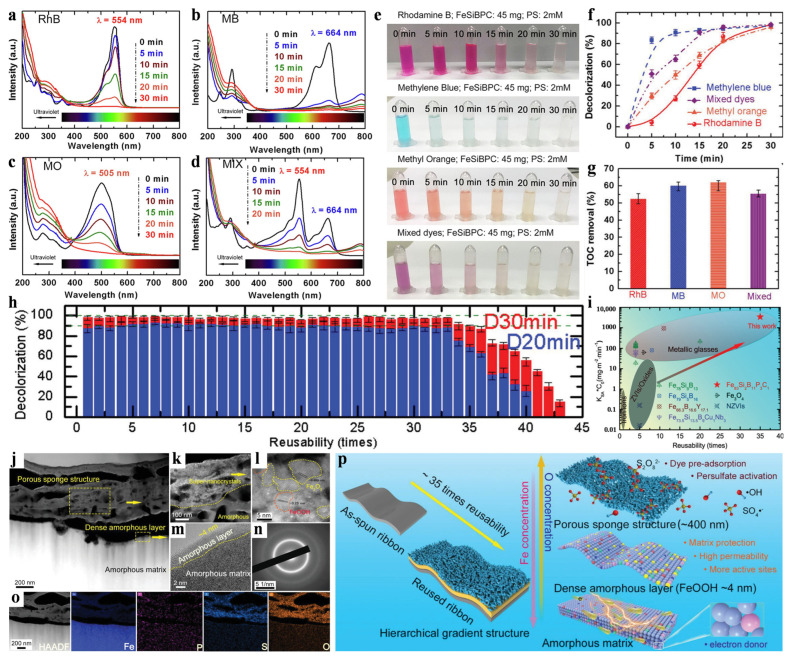
UV–Vis spectra of (**a**) RhB, (**b**) MB, (**c**) MO, and (**d**) mixed dye. (**e**) Visible color of various dyes. (**f**) Decolorization efficiency. (**g**) TOC removal using Fe_83_Si_2_B_11_P_3_C_1_ catalyst. (**h**) Reusability of Fe_83_Si_2_B_11_P_3_C_1_ catalysts. (**i**) Degradation capability vs. reusability of various catalysts. STEM-HAADF images of (**j**) the cross-sectional structure and (**k**,**l**) the porous sponge area. (**m**) HRTEM image of the dense amorphous layer in (**j**). (**n**) SAED image of (**m**). (**o**) Elemental mapping of Fe, P, S, and O. (**p**) The catalytic reaction mechanism of Fe_83_Si_2_B_11_P_3_C_1_ catalysts. Reproduced with permission [47]. Copyright 2019 WILEY-VCH Verlag GmbH & Co. KGaA, Weinheim, Germany.

The catalytic degradation of a dye solution by activating the H_2_O_2_ in Fe-based metallic glass is described above. Now, the catalytic capacity and mechanism of the persulfate system are reviewed. Figure 6a–d present examples of the UV–Vis spectra of rhodamine B (RhB), methylene blue (MB), methyl orange (MO), and mixed dye decolorization. Notably, with the increase in reaction time, the absorption peaks of each dye at 554 nm, 664 nm, 505 nm and 554 nm, respectively, show a decreasing trend. This indicates that the dye solutions have been effectively decolorized. Figure 6e shows the color decolorization of various dyes at a concentration of 20 ppm under the conditions of the persulfate system. This reaction system only requires the addition of 2 mM persulfate and 45 mg of Fe_83_Si_2_B_11_P_3_C_1_ glass ribbons, which indicates that it has broad application prospects in the remediation of industrial wastewater. Fe_83_Si_2_B_11_P_3_C_1_ metallic glass catalyst removed nearly 100% of the color and more than 50% of the TOC in 30 min of four different dye solutions (Figure 6f,g). In a repeatability test, a metallic glass catalyst with Fe_83_Si_2_B_11_P_3_C_1_ as the atomic component had 35 times the reusability in the degradation of RhB solution (Figure 6h). In order to further highlight the excellent catalytic performance of Fe_83_Si_2_B_11_P_3_C_1_ metallic glass catalyst, Figure 6i summarizes a comparison of the results of the degradation ability and the reusability of amorphous and crystalline Fe-based catalysts. The stability and efficiency of ZVIs and Fe-based oxides are greatly limited due to the influence of crystal structure defects. A crystalline-iron-based Fenton catalyst was reported to be reusable less than 10 times. Compared with the crystalline catalyst, the Fe-based glass catalyst has higher catalytic capacity and stability in the degradation of dye wastewater. It is worth noting that Fe_83_Si_2_B_11_P_3_C_1_ metallic glass ribbon provides some of the best comprehensive performance.

In order to demonstrate the structural evolution of the Fe_83_Si_2_B_11_P_3_C_1_ metallic glass catalyst after repeated use, its cross-sectional structure was characterized using spherical aberration corrected HAADF-STEM, as shown in Figure 6j–o. Notably, the surface of the reused metallic glass catalyst evolves into a layered structure consisting of a top porous sponge layer (~500 nm), a thin amorphous layer (~4 nm), and an amorphous matrix. The evolution of this microstructure provides significant improvements in persulfate activation and organic degradation. In addition, adding certain elements to metallic glass catalysts can improve their surface stability. Figure 6p shows a schematic diagram of the effects of surface and structural changes on the catalytic reaction mechanism of Fe_83_Si_2_B_11_P_3_C_1_ metallic glass catalyst. The hierarchical gradient structure consisting of a porous sponge layer and a dense amorphous thin layer is gradually self-reconstructed under catalytic degradation with repeated use [23,54]. Persulfate molecules quickly pass through the porous channel of the sponge surface to reach the amorphous interlayer and are activated by ^•^OH and SO_4_^•−^, which is conducive to the effective catalysis of organic dye molecules. The densely arranged thin amorphous interlayer and stable amorphous matrix have excellent catalytic activity and stability, which guarantee for the catalytic degradation process [55]. The catalytic reactions in the persulfate system include the following [56,57]:

Fe^0^ + S_2_O_8_^2−^ → Fe^3+^ + SO_4_^−•^ + SO_4_^2−^ + 2e^−^ (5)

SO_4_^−•^ + H_2_O → SO_4_^2−^ + ^•^OH + H^+^ (6)

^•^OH/SO_4_^−•^ + Organic → Products (7)

**Figure 7:**
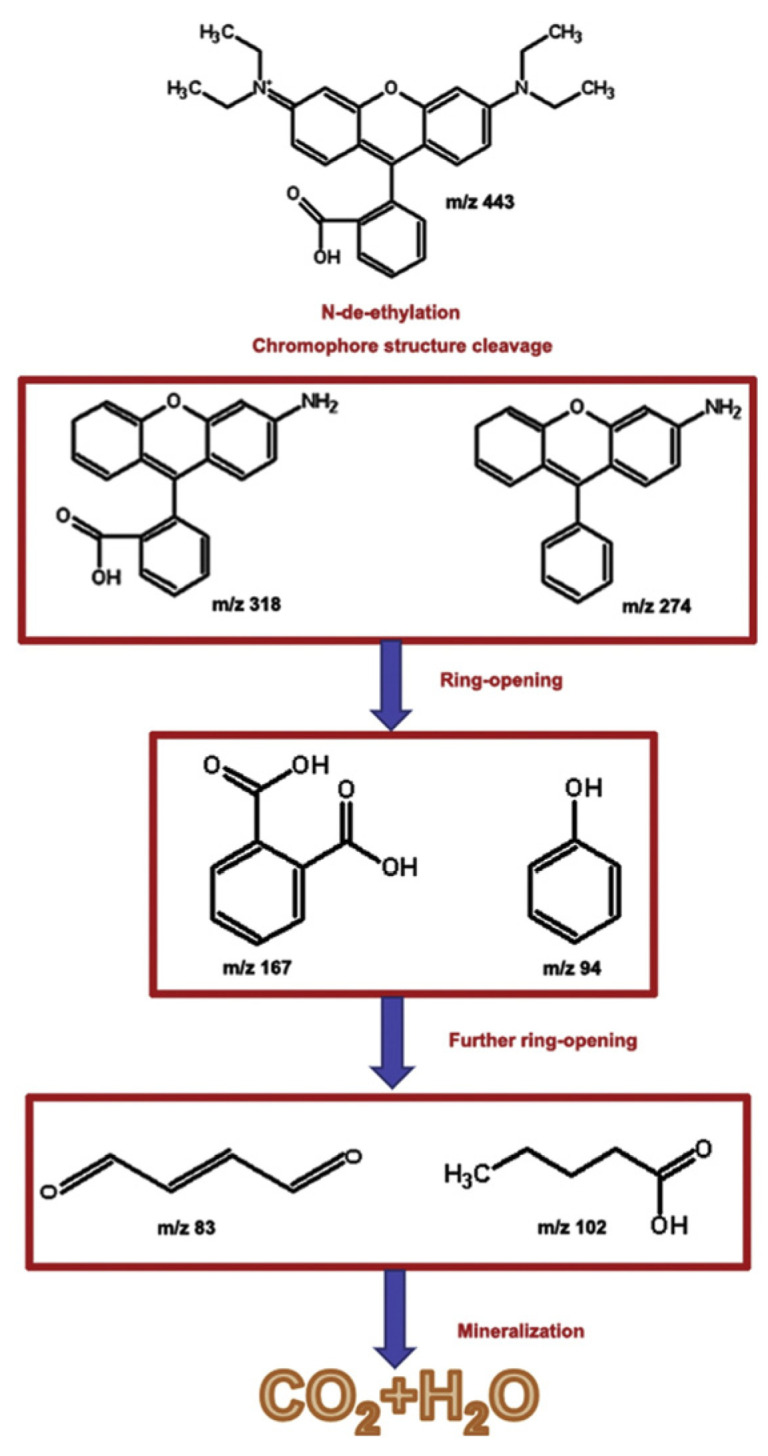
Proposed degradation pathways of RhB by SO_4_^•−^ radicals. Reproduced with permission [58]. Copyright 2020 Elsevier Ltd.

Figure 7 shows the degradation pathways of RhB dye under the action of SO_4_^•−^ groups. First, the methyl groups are shed from RhB molecules via SO_4_^•−^ attack, resulting in a de-ethylation product. Second, when the organic matter is further degraded, the chromophore structure’s bond between the anthracene group and phenyl group is destroyed, resulting in RhB decolorization. Third, the ring-opening process produces low-molecular-weight acids, which are further mineralized.

### 3.2. Energy Catalyst

**Figure 8:**
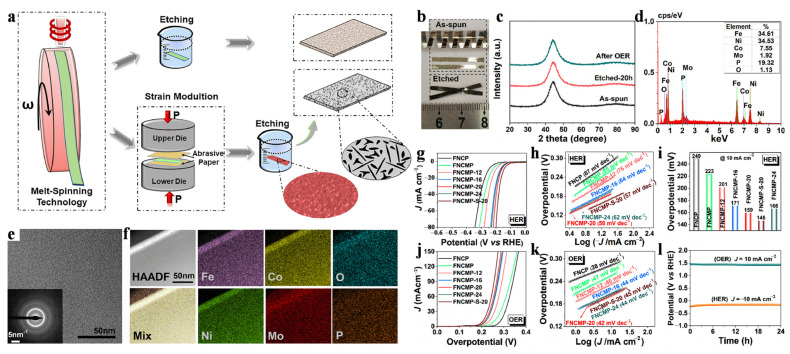
(**a**) The preparation process of FNCMP samples. (**b**) Macrograph of FNCMP. (**c**) XRD patterns and (**d**) EDS of FNCMP. (**e**) TEM image and (**f**) elemental mapping. The inset of (**e**) shows the SAED pattern. (**g**) HER LSV curves and (**h**) Tafel plots of FeNiCo(Mo)P. (**i**) The overpotential, (**j**) OER LSV curves, and (**k**) Tafel plots of the samples. (**l**) Chronopotentiometric curves of an FNCMP-20 sample. Reproduced with permission [59]. Copyright 2021 Elsevier Ltd.

The morphology of modulated Fe_35_Ni_35_Co_8_Mo_2_P_20_ (FNCMP) samples is schematically illustrated in Figure 8a, where two different preparation methods are shown: (i) as-spun ribbons etched alone in an acidic solution and (ii) as-spun ribbons etched in acidic solutions after strain modulation. Figure 8b optical photograph shows the actual morphology and flexibility of amorphous FNCMP ribbons. XRD patterns show that the samples in different states have the typical wide diffraction peaks, which confirms their amorphous structure (Figure 8c). EDS results show that the composition of the material corresponds to the expected composition (Figure 8d). TEM images show a disordered atomic structure, and the corresponding selected electron diffraction spectrum reconfirms the amorphous structure (Figure 8e). The element mapping shows the homogeneous distribution of Fe, Ni, Co, Mo, and P in the amorphous FNCMP ribbon (Figure 8f), as well as a trace amount of O, verifying the composition homogeneity of the prepared sample. FNCMP samples are etched for different durations, for example, 12, 16, 20, or 24 h. The prepared samples are used as catalysts for hydrogen evolution reaction (HER) and oxygen evolution reaction (OER) performance tests.

The HER activity of these prepared metallic glass catalysts in 1 M NaOH solution is shown in Figure 8g–i. Compared with that of the FeNiCoP (FNCP) catalyst, the HER of the FNCMP catalysts has an overpotential of *η*_10_ = 223 mV and a Tafel slope of 85 mV dec^−1^. The etching results showed that the HER activity of the FNCMP catalyst increased gradually from 12 h to 20 h but decreased slightly when etching time increased to 24 h. Notably, the FNCMP-20 catalyst has an exceptional overpotential of *η*_10_ = 159 mV and a Tafel slope of 58 mV dec^−1^. In addition, the OER electrocatalytic activities of FNCMP catalysts are shown in Figure 8j. The results show that the overpotential of the FNCMP catalyst is 256 mV, which is lower than the overpotential of the as-spun FNCP catalyst of 281 mV, indicating that the Mo can effectively improve the alkaline OER activity of the metallic glass catalyst. Similarly, with the increase in etching time, the change in the OER activity of the FNCMP catalyst in 1 M NaOH solution is similar to that in the HER. The corresponding OER kinetics increased with the increase in etching treatment time. The Tafel slopes of FNCMP-12, FNCMP-16, FNCMP-20, and FNCMP-24 catalysts are approximately 46 mV dec^−1^, 44 mV dec^−1^, 42 mV dec^−1^, and 44 mV dec^−1^, respectively (Figure 8k). Compared with other metallic glass catalysts, the FNCMP-20 catalyst has the best OER performance. In addition to its electrocatalytic activity, the long-term stability of the HER and OER of FNCMP-20 catalysts at current densities of −10 and 10 mA cm^−2^ was also analyzed via chronopotentiometry, showing excellent stability over a 24 h duration (Figure 8l). The above review shows that the electrocatalytic activity of metallic glass can be significantly improved after a certain period of etching, which proves the reliability of the etching process.

**Figure 9:**
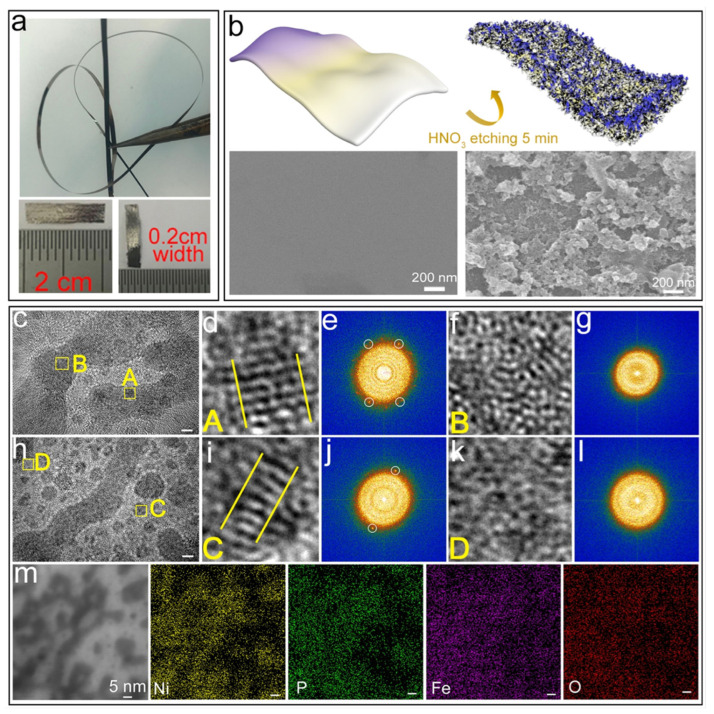
(**a**) Macrograph of FeNiPC ribbon. (**b**) Surface morphological evolution of FeNiPC ribbon. (**c**,**h**) HRTEM images of acid-treated FeNiPC ribbon. (**d**,**f**,**i**,**k**) HRTEM images of A, B, C, and D regions from (**c**,**h**). (**e**,**g**,**j**,**l**) Corresponding FFT patterns of the regions in (**d**,**f**,**i**,**k**), respectively. (**m**) HAADF-STEM image and elemental mapping of acid-treated FeNiPC ribbon. Reproduced with permission [28]. Copyright 2022 American Chemical Society.

In order to explain the mechanism through which etching enhances the electrocatalytic activity and the evolution of the metallic glass microstructure, the catalytic performance of the Fe_50_Ni_30_P_13_C_7_ (FeNiPC) metallic glass catalyst is reviewed. Figure 9a shows the macro form of FeNiPC metallic glass ribbon. Figure 9b shows a schematic diagram of the surface structure and evolution process using FeNiPC metal glass strips as precursors. It is worth noting that after 5 min of acid treatment, the original surface morphology of as-spun metallic glass ribbon changed from ultra-smooth to microplate-like. Figure 9c–m show the microstructure evolution of the catalyst surface after treatment with FeNiPC metallic glass acid for 5 min. Figure 9c,h show a typical HRTEM image of the uppermost layer of the FeNiPC catalyst’s surface, indicating that its structure is mainly amorphous. However, it is worth noting that the A and C regions in Figure 9c,h present a clear local atomic order. The corresponding amplification region (Figure 9d,i) shows that this ordered arrangement of atoms is consistent with the (021) plane of the crystal Ni_2_P phase. The FFT pattern of the region in Figure 9e,j shows no ordered lattice, which confirms the existence of nanocrystals. However, the disordered atomic structures and amorphous diffracting halos are evident in regions B and D, indicating that these regions have amorphous structures (Figure 9f,g,k,l). Figure 9m shows the HAADF-STEM image and element mapping results of the FeNiPC catalyst. The results show that Ni and P are mainly distributed in the dark region, while Fe and O are mainly concentrated in the bright region. In addition, in area B in Figure 9f, the results show that the dark region of the FeNiPC catalyst contains both the Ni_2_P crystalline phase and the Ni_2_P amorphous phase. Combined with the structure analysis of region D in Figure 9k, it can be seen that the bright region of the FeNiPC catalyst mainly includes FeOOH. Finally, the structural characterization results show that the surface of the FeNiPC catalyst after acid treatment mainly consists of crystalline Ni_2_P, amorphous Ni_2_P, and amorphous FeOOH catalytic active sites.

**Figure 10:**
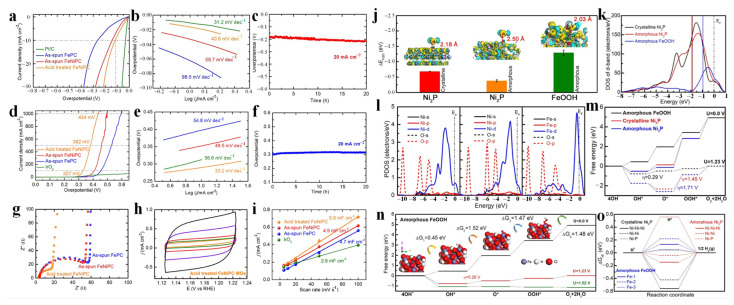
(**a**) HER polarization curves, (**b**) Tafel slopes, (**c**) stability performance of ribbon samples. (**d**) OER polarization curves, (**e**) Tafel slopes, (**f**) stability performance of ribbon samples. (**g**) EIS measurements of amorphous ribbons. (**h**) Cyclic voltammetry curves measured at various scan rates in the range of 5–100 mv s^−1^. (**i**) Capacitive currents. (**j**) DFT calculation of Δ*E*_H2O_. (**k**) *d*-PDOS and (**l**) PDOSs of the three models. Free-energy profiles of the (**m**) three models and (**n**) amorphous FeOOH. (**o**) Gibbs free-energy (Δ*G*_H*_) profiles. Reproduced with permission [28]. Copyright 2022 American Chemical Society.

The water oxidation activity of prepared flexible independent metallic glass catalyst in 1.0 M KOH solution was investigated. In terms of HER properties, the prepared acid-treated FeNiPC catalyst shows excellent catalytic activity compared with that of as-spun metallic glass and benchmark Pt/C nanoparticles, as shown in Figure 10a–c. At a current density of 10 mA cm^−2^, the overpotential of the acid-treated FeNiPC catalyst is 113 mV, which is better than that of the as-spun FeNiPC (143 mV) and FePC (262 mV) ribbons (Figure 10a). The slope of the HER Tafel obtained with acid-treated FeNiPC catalyst is 40.6 mV dec^−1^ (Figure 10b), which is also significantly higher than that of as-spun metallic glass and close to that of Pt/C nanoparticles. In addition, under alkaline conditions, the acid-treated FeNiPC catalyst also shows good HER stability over 20 h (Figure 10c). Figure 10d shows the OER polarization curve of the metallic glass catalyst at a scanning rate of 5 mV s^−1^. The overpotential of the FeNiPC catalyst is 352 mV, which is much lower than FePC, indicating that Fe and Ni have a synergic effect on the enhancement in OER activity. After the acid-treatment process, the overpotential of the FeNiPC catalyst drops sharply to 289 mV, which is much lower than the overpotential of industrial IrO_2_ particles (320 mV). In addition, the Tafel slope value of the FeNiPC catalyst reaches ~33.2 mV dec^−1^ (Figure 10e), which is also significantly lower than that of as-spun and IrO_2_ catalysts. Figure 8f shows the OER stability of the FeNiPC catalyst recorded according to chronoammetry. Notably, the FeNiPC catalyst can be stabile for more than 20 h. Figure 10g shows the EIS measurement results of each catalyst in 1.0 M KOH solution. The semicircle of the acid-treated FeNiPC catalyst is smaller than that of the as-spun catalysts, indicating that its electron transfer ability is stronger. The C_dl_ is determined via the cyclic voltammetry method (Figure 10h). Notably, the C_dl_ of the acid-treated FeNiPC catalyst is 5.9 mF cm^−2^, which is higher than the C_dl_ value of the as-spun and IrO_2_ catalysts (Figure 10i). The catalytic activity of the FeNiPC catalyst is higher than that of the original catalysts. The results show that the hydroxylation of the catalyst surface increased the number of active sites and finally improved the reaction kinetics.

In order to reveal the mechanism of its water decomposition activity, DFT calculations were performed to achieve an understanding of the atomic structure of the FeNiPC catalyst. Atomic models of crystalline Ni_2_P, amorphous Ni_2_P, and amorphous FeOOH were established to study their water-splitting properties. The Δ*E*_H2O_ values of various active sites are shown in Figure 10j. The results show that FeOOH has the highest Δ*E*_H2O_ value of −1.29 eV, while crystalline Ni_2_P and amorphous Ni_2_P have Δ*E*_H2O_ values of −0.68 and −0.38 eV, respectively. The inset in Figure 10j shows the difference in electron density after adsorption of the three models. The results show that the charge transferability of amorphous FeOOH and H_2_O is the strongest, followed by that of crystalline Ni_2_P, and that of amorphous Ni_2_P is the weakest. In addition, the equilibrium distances of the active sites of crystalline Ni_2_P, amorphous Ni_2_P, and amorphous FeOOH are 2.18, 2.50, and 2.03 Å, respectively. To investigate the effect of binding strength, the *d*-band center and PDOS between H_2_O and the three models were calculated, as shown in Figure 10k,l. The *d*-band center of FeOOH is closer to E_F_ than that of crystalline and amorphous Ni_2_P (Figure 10k). In addition, the electron interaction between the d orbitals of Fe and the p orbitals of O in FeOOH is much stronger than the electron interaction between the d orbitals of Ni and the p orbitals of O (Figure 10l). This suggests that amorphous FeOOH plays a major role in the H_2_O adsorption process. Figure 10m shows the free-energy distribution of OER processes under zero and equilibrium potentials for the three models. Notably, the Δ*G*_2_ of amorphous FeOOH is ~1.52 eV, and the theoretical overpotential (*η*) is 0.29 V. The Δ*G*_3_ values of crystalline and amorphous Ni_2_P are 2.68 and 2.94 eV, respectively, and the *η* values are 1.45 and 1.71 V, respectively. The lower overpotential required for FeOOH indicates the excellent OER properties of its atomic configuration. In addition, when the overpotential increases to 1.52 V, the basic reaction steps for FeOOH are all downhill reactions (Figure 10n). Figure 10o shows the Δ*G*_H*_ at different catalytic sites. The Δ*G*_H*_ values of the six catalytic sites are all less than ± 0.20 eV, and the Δ*G*_H*_ values of three sites being all less than ±0.10 eV indicates that the structural heterogeneity of metallic glass ribbons contributes significantly to their HER activity. The DFT results show that FeOOH is the most complete electrocatalytic active configuration, which is similar to the results of other studies [55]. In addition, the degradation rate (*k*) of Fe-based metallic glasses in dye wastewater, the overpotential, and Tafel slope of Fe-based metallic glasses during water splitting are compared in Table 2.

## 4. Conclusions and Prospects

In this review article, we summarized the common methods of preparing metallic glass catalysts and the current research on catalysts in the environment and energy fields. Because of the unique atomic configuration of metallic glass, its preparation is more complicated than that of metallic crystal. As a new type of catalyst, metallic glass has shown excellent catalytic performance in the field of dye wastewater purification and in water splitting due to its excellent chemical reactivity and sustainability. In addition, many scholars have found that nanoscale construction on the surface of the original metallic glass catalyst can effectively increase the number of surface active sites and thus significantly improve its catalytic activity. However, in order to realize the industrialization of metallic glass catalysts in water environment treatment and water splitting, several important theoretical and technical problems from the preparation to the understanding of the reaction mechanism of metallic glass catalysts need to be solved: First, the scale of the preparation methods of metallic glass catalyst need to be expanded, the cost needs to be reduced, and the difficulty of operation must be simplified. Second, the water environment for catalysis should be improved so that the catalyst has a wider pH range and be applied to all water environments to reduce the addition of additives. Finally, a perfect reaction mechanism system needs to be established to clarify the internal relationship between catalytic activity and atomic structure on the catalyst surface. In view of some key problems facing the development of metallic glass, we believe that the first problems to be solved is the production cost; nonprecious metals should be used to avoid the use of precious metal elements to guarantee the amorphous formation ability by ensuring a reasonable atomic proportion, and the production steps need to be reduce. As for how to make the catalyst suitable for more water environments and to establish a reaction mechanism system, it is necessary to collect experimental data, understand the catalytic characteristics of different amorphous alloy systems, and theoretically prove and predict the amorphous system in combination with theoretical simulation calculations, so as to establish an effective correlation between experiments and theory. In summary, through the continuous research on Fe-based metallic glass catalysts, as well as clarification and improvement of catalytic mechanism, we believe that the catalytic activity of metallic glass can be improved, and their practical application will be on the near horizon.

## Figures and Tables

**Table 1:** Comparison of preparation of amorphous alloys via melt-spinning, ball-milling, and gas-atomization methods.

Method	Mechanism	Morphology	Specific Surface Area	Preparation Cycle	Refs.
Melt spinning	Rapid cooling	Smooth ribbon	Low	Fast	[39,43]
Ball milling	Mechanical crushing	Rough powder	High	Slow	[20,44]
Gas atomization	Rapid cooling	Smooth powder	Medium	Fast	[19,20]

**Table 2:** Comparison of the properties of various Fe-based metallic glasses in wastewater treatment and during water splitting.

Metallic Glass (Environment)	Organic Pollutant	Initial Concentration (mg·L^−1^)	*k* (min^−1^)	Ref.
Fe_78_Si_9_B_13_	Methyl blue	20	0.381	[50]
Fe_78_Si_9_B_13_	Blliant red 3B-A	50	0.654	[52]
Fe_78_Si_9_B_13_	Methyl blue	20	0.356	[41]
Fe_78_Si_9_B_13_	Brilliant Yellow 3G-P	20	0.466	[41]
Fe_78_Si_9_B_13_	Brilliant red 3B-A	20	0.372	[41]
Fe_78_Si_9_B_13_	Malachite green	20	0.519	[41]
Fe_78_Si_9_B_13_	Malachite green	20	0.057	[41]
Fe_78_Si_9_B_13_	Methylene blue	20	0.64	[46]
Fe_78_Si_13_B_9_	Direct blue 6	200	0.115	[60]
Fe_78_Si_13_B_9_	Orange II	100	0.238	[60]
Fe_78_Si_13_B_9_	Methyl orange	25	0.103	[60]
Fe_78_Si_9_B_13_	Methylene blue	20	0.302	[61]
Fe_78_Si_9_B_13_	Reactive red 195	100	0.495	[17]
Fe_78_Si_9_B_13_	Methylene blue	100	0.37	[62]
Fe_80_P_13_C_7_	Methylene blue	100	0.56	[62]
Fe_78_(Si,B)_22_	Orange II	100	0.125	[63]
Fe_78_Si_8_B_14_	Acid orange II	200	0.174	[64]
Fe_79_B_16_Si_5_	Orange G	100	0.004	[65]
Fe_66.3_B_16.6_Y_17.1_	Orange G	100	0.047	[65]
Fe_75_P_15_C_10_	Reactive red 195	100	0.307	[39]
(Fe_0.99_Mo_0.01_)_78_Si_9_B_13_	Acid orange II	100	0.168	[66]
Fe_73.5_Si_13.5_B_9_Cu_1_Nb_3_	Methyl orange	20	0.152	[50]
Fe_73.5_Si_13.5_B_9_Cu_1_Nb_3_	Methyl blue	20	0.201	[50]
Fe_73.5_Si_13.5_B_9_Cu_1_Nb_3_	Methylene blue	20	0.119	[61]
Fe_83_Si_2_B_11_P_3_C_1_	Rhodamine B	20	0.09	[47]
Fe_83_Si_2_B_11_P_3_C_1_	Rhodamine B	20	0.36	[47]
Fe_83_Si_2_B_11_P_3_C_1_	Rhodamine B	20	0.165	[47]
**Metallic Glass** **(Energy)**	**Electrolyte Solution**	**Overpotential (mV)**	**Tafel Slope (mV dec^−1^)**	**Ref.**
Fe_35_Ni_35_Co_8_Mo_2_P_20_ (HER)	1 M NaOH	159	58	[59]
Fe_35_Ni_35_Co_8_Mo_2_P_20_ (OER)	1 M NaOH	213	42	[59]
Fe_50_Ni_30_P_13_C_7_ (OER)	1 M KOH	327	33.2	[28]
Fe_50_Ni_30_P_13_C_7_ (HER)	1 M KOH	113	40.6	[28]
(Fe_73.5_Si_13.5_B_9_Nb_3_Cu_1_)_91.5_Ni_8.5_ (OER)	1 M KOH	395	100	[67]
Fe_73_C_3_Si_7.3_B_8.5_P_5.7_Mo_2.5_ (HER)	1 M KOH	476	115	[68]
Fe_59.5_C_3_Si_7.3_B_8.5_P_5.7_Mo_2.5_Co_13.5_ (HER)	1 M KOH	462	110	[68]
Fe_40_Co_35_Mo_5_P_13_C_7_ (HER)	0.5 M H_2_SO_4_	90	42	[69]

## Data Availability

Data are contained within the article.
